# Dutch–Flemish translation and validation of the gastrointestinal symptom scales from the patient‑reported outcomes measurement information system (PROMIS)^®^

**DOI:** 10.1186/s41687-023-00662-z

**Published:** 2023-11-30

**Authors:** Mirjam van der Ende-van Loon, Dorinde Korteling, Hilde Willekens, Monique Schilders, Wouter Curvers, Raf Bisschops, Erik Schoon, Caroline Terwee

**Affiliations:** 1 Department of Gastroenterology and Hepatology, Catharina Hospital, Michelangelolaan 2, 5623 EJ Eindhoven, The Netherlands; 2 Child and Adolescent Psychiatry and Psychosocial Care, Amsterdam UMC Location, Emma Children’s Hospital, Amsterdam, The Netherlands; 3 Amsterdam Reproduction and Development, Child Development, Amsterdam, The Netherlands; 4 Amsterdam Public Health, Mental Health and Methodology, Amsterdam, The Netherlands; 5 Department of Gastroenterology and Hepatology, University Hospitals Leuven, Leuven, Belgium; 6 GROW: School for Oncology and Developmental Biology, Maastricht University, Maastricht, The Netherlands; 7 Department of Epidemiology and Data Science, Amsterdam UMC, Vrije Universiteit Amsterdam, Amsterdam, The Netherlands; 8 Amsterdam Public Health Research Institute, Methodology, Amsterdam, The Netherlands

**Keywords:** Patient-reported outcomes measurement information system (PROMIS), Gastrointestinal symptoms, Translation, Validation, PROs, PROM

## Abstract

**Purpose:**

To translate the eight PROMIS^®^ GastrointestinaI Symptom Scales into Dutch–Flemish and to evaluate their psychometric properties.

**Methods:**

This study consisted of two parts: (1) translation according to the Functional Assessment of Chronic Illness Therapy (FACIT) translation methodology and (2) evaluation of psychometric properties: structural validity, using confirmatory factor analysis; and construct validity using hypothesis testing.

**Results:**

In the first part of the study, in 19 out of the 77 items (24.7%) translation was challenging. After discussion between the translators, consensus could be achieved. In the cognitive debriefing interview phase, ten minor changes in the wording of items were made. A universal Dutch–Flemish translation for all 77 items was obtained. In de second part of the study a good fit was found for three DF-PROMIS GI Scales: Bowel Incontinence, Gas and Bloating, and Belly Pain. Four scales (Reflux, Disrupted Swallowing, Diarrhea, and Constipation) did not show sufficient fit and fit for the Nausea and Vomiting scale could not be assessed because of skewed responses. Construct validity was considered sufficient for six out of eight DF-PROMIS GI Scales. Less than 75% of hypothesis for de Constipation and Disrupted Swallowing scales could be confirmed.

**Conclusion:**

The PROMIS GI Symptom Scales were successfully translated into DutchFlemish. The findings suggest a sufficient structural validity for the PROMIS GI Scales. Bowel Incontinence, Gas and Bloating and Belly Pain. Construct validity was sufficient for the Scales Gas and Bloating, Incontinence, Nausea and Vomiting, Reflux, Belly Pain, and Diarrhea.

## Introduction

Gastrointestinal (GI) symptoms are widespread and bring substantial economic and social consequences. The prevalence of gastrointestinal diseases in Western countries has increased over the past few decades and is one of the most commonly encountered conditions in primary care practice. A large-scale multinational study, found that more than 40% of persons worldwide have functional *gastrointestinal disorders* (FGID). Data from the Netherlands show a prevalence of 30.6% and 35.6% in Belgium. Functional constipation and IBS were most prevalent [1]. Individuals with any FGID showed lower global physical health and global mental health, as measured with the PROMIS^®^ Global Health Scale, compared with subjects with no FGID, which affects quality of life and increases health care use [1].

The importance of patients’ perspectives on the impact of disease and response to treatment is widely recognized. Patient-reported outcome measures (PROMs) measure the patient's health status from the patient’s perspective. For measuring patients’ perspectives on GI symptoms, over the past 2 decades investigators have developed over 100 disease-targeted PROMs [2]. However, scores from these different questionnaires are not comparable since they utilize different measurement scales. Furthermore, it is often unclear which changes in scores are relevant in daily practice. It is important to standardize outcome measurements and use the same PROMs as much as possible across all GI disorders for clinical and research purposes.

The eight National Institutes of Health (NIH) PROMIS GI Symptom Scales capture GI symptoms experienced by people with a wide range of digestive disorders. Unlike disease-targeted measures, which are designed for specific patient populations, the PROMIS-GI Symptom Scales are system-targeted measures, designed for anyone experiencing GI symptoms, whether patients or members of the population at large [3]. This is an important unique value of PROMIS measures, because disease-targeted PROMs are not useful across the population as a whole [3, 4]. The original PROMIS-GI Symptom Scales were developed by Spiegel et al. in the Unites States of America. The scales correlated significantly with both generic and disease- targeted legacy instruments, and demonstrate evidence of reliability [3]. The PROMIS-GI symptom scales can be used together or individually in clinical practice and clinical research and are broadly applicable across populations, GI symptoms, GI diseases, and demographics. The PROMIS GI symptom Scales have been translated and validated in different languages, however there is no data published yet on the psychometric properties of these translations.

By translation of the PROMIS Gastrointestinal Symptoms Scales into Dutch–Flemish we will make these instruments available for use in the Netherlands and Flanders (the Dutch-speaking part of Belgium) in patients with a broad range of GI diseases. This study aimed to translate the PROMIS Gastrointestinal Symptom Scales into Dutch–Flemish and to evaluate their psychometric properties structural validity and construct validity in patients with a variety of GI conditions.

## Methods

This study consisted of two parts: (1) translation of the PROMIS-GI Scales v1.0 into Dutch–Flemish (DF) and (2) evaluation of psychometric properties structural validity using confirmatory factor analysis (CFA) and construct validity using hypothesis testing in GI patients. Authorization to translate the eight PROMIS GI Symptom Scales was obtained from the Health Measures translation team in June 2021. For both parts of this study, patients were recruited from the Catharina Hospital in the Netherlands and the University Hospital UZ Leuven in Belgium. Patients were eligible if aged 18 years, and confirmed diagnosis of Inflammatory bowel disease (IBD), irritable bowel syndrome (IBS) or gastroesophageal reflux disease (GERD) with or without a Barrett’s esophagus, had to be able to read, understand and complete the Dutch informed consent form and the study questionnaires. Informed consent was obtained from all participants.

### Translation and cognitive debriefing

**Fig. 1:**
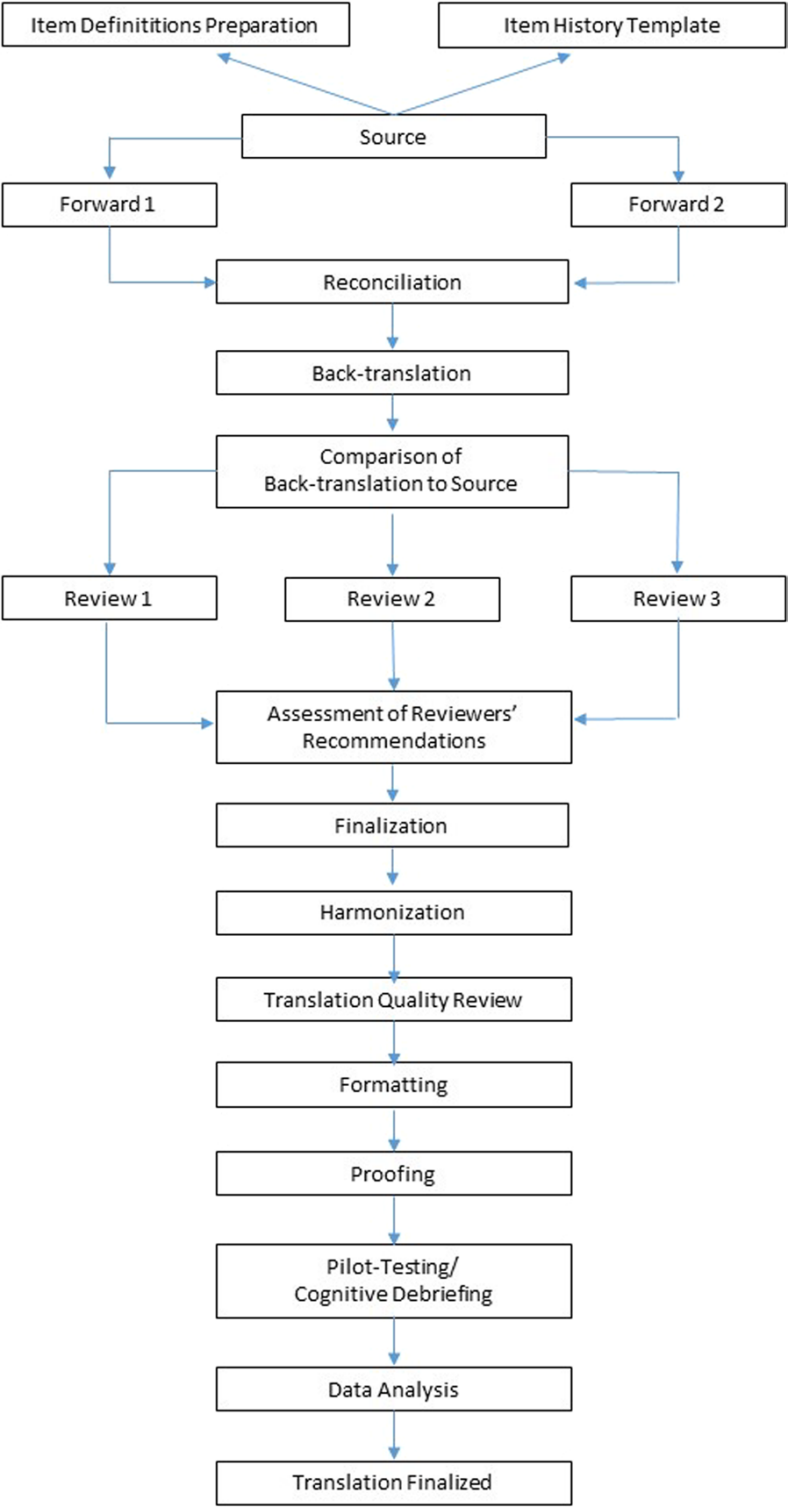
FACIT translation methodology chart

The translation process followed the Functional Assessment of Chronic Illness Therapy (FACIT) translation methodology [5]. The steps of the FACIT translation methodology included two forward translations (by 1 Dutch and 1 Flemish native-speaker), and one backward translation (English native-speaker), independent review by two reviewers (ME and CT), harmonization with previous PROMIS translations and assessment of translation quality by the Dutch–Flemish PROMIS National Center (CT), and pilot testing including cognitive debriefing (Fig. 1).

To assess comprehensibility, cognitive debriefing interviews were performed with 10 native Dutch-speaking participants in the Netherlands and 10 native Flemish-speaking participants in the Flemish-speaking part of Belgium. Participants included five persons from the general population and five patients with GI symptoms in each country. Participants from the general population were recruited from the social network of employees working in the GI department of the two hospitals. Participants were selected based on age, gender, education level, and disease to obtain heterogeneity in the population sample. Informed consent was obtained from all participants. The interview script was based on the retrospective verbal prompting technique, following prior PROMIS work [6]. During the interviews, participants first completed all translated items in writing. Subsequently, participants were asked about difficulties in understanding each item and the meaning of the items were discussed to ensure comprehensibility. After completing the interviews with 10 Dutch participants, some adjustments were made to the translations of the response categories and items. Thereafter, another 10 interviews were completed with participants from Belgium to test the modified versions of the items. All interviews were audio recorded.

### Psychometric testing

The aim of the psychometric testing phase was assessing structural validity and construct validity of the DF-PROMIS GI Symptom Scales using a cross-sectional study design in patients with GI conditions. For assessing construct validity, all patients completed the DF-PROMIS GI questionnaire Gastrointestinal Symptom Rating Scale (GSRS). In addition, Inflammatory Bowel Disease (IBD) patients completed the Inflammatory Bowel Disease Questionnaire (IBDQ) and Irritable Bowel Syndrome (IBS) patients completed the Irritable Bowel Syndrome Quality of Life Questionnaire (IBS-QOL).

For validation purposes, COSMIN guidelines recommend a sample of 7 times the number of items per scale and at least 100 for a study of very good quality [7]. Questionnaires were completed at home, with a postal or digital return of the questionnaire in Research manager (version 5.2.2).

### Measurements

Patients were asked to fill out several demographic and clinical questions (age, sex, and educational level).

#### PROMIS GI

The DF-PROMIS GI Symptom Scales consist eight scales: Reflux (13 items), Disrupted Swallowing (7 items), Diarrhea (5 items), Bowel Incontinence (4 items), Nausea and Vomiting (4 items), Constipation (9 items), Belly Pain (6 items), and Gas and Bloating (12 items). The PROMIS GI scales can be used individually or in combination and are subsequently scored and reported individually. All items, except for one, are administered using a 5-point categorical response scale. The first item in Gas and Bloating is an unscored item (GISX94). Its response options are “A = yes” and “B = no” and do not contribute to the summed score. There were expected missing responses on items in the Scales Reflux, Diarrhea, Bowel Incontinence, Nausea and Vomiting, Belly Pain, and Gas and Bloating. These scales contain response instructions with “if never, go to…” As a result, patients without symptoms skipped one or more items.

For all scales, except the Bowel Incontinence Scale, T-scores were calculated using the response pattern scoring service available at the Health Measures website. T-scores were based on the underlying Item-response theory (IRT) models. IRT models are used for establishing whether a set of items intended to measure a particular attribute, together constitute a scale for measurement [8].

Higher T-scores indicate more symptoms. Each GI scale was calibrated by the original developers using a IRT graded response model and IRT scores were converted to T scores with a mean of 50 and SD of 10 in the US general population, by PROMIS convention [11]. However, previous studies did not produce an IRT based T-score for the Bowel Incontinence scale. Therefore, simple summed scores for this scale were used in analysis.

#### The gastrointestinal symptom rating scale (GSRS)

The GSRS is a 15-item questionnaire that evaluates the five common symptom clusters of Gl disorders: abdominal pain, reflux, indigestion, constipation and diarrhea [9]. Items ask about the past week using a 7-point categorical response scale ranging from no discomfort to very severe discomfort. The self-administered version of the GSRS utilized in this study showed an acceptable reliability, validity, and responsiveness to change in patients with different GI disorders [10, 11]. The GSRS has five-symptom domains representing reflux, abdominal pain, indigestion, diarrhea and constipation. A score for each domain was calculated based on the average score of the questions in that domain with higher scores indicating more symptoms.

In addition to completing the DF-PROMIS GI Scales and the GSRS, patients completed a relevant disease-targeted legacy instrument: IBS patients completed the IBS-QOL, IBD patients completed the IBDQ.

#### Irritable bowel syndrome quality of life questionnaire (IBS-QOL)

The IBS-QOL is a well-established 34-item measure assessing the degree to which IBS interferes with a patient’s quality of life. Each item is rated on a 5-point Likert scale, ranging from not at all to extremely or a great deal, yielding a total score that ranges from 34 to 170 [12, 13]. As per the IBS-QOL scoring manual, all items were reversed and raw summary scores were transformed into a 0 to 100 scale with higher scores indicate better QOL [14].

#### Inflammatory bowel disease questionnaire (IBDQ)

The validated Dutch version of the IBDQ was used in IBD patients. The IBDQ is a 32-item questionnaire assessing bowel symptoms, systemic symptoms, emotional function, and social function. All items use 7-point Likert scales for capturing symptom-related experiences during the past 2 weeks, where 1 represents the highest symptom frequency/severity and 7 indicates the lowest symptom frequency/severity. The total score ranges from 32 (poor quality of life) to 224 (good quality of life). IBDQ total score higher than 170 is usually associated with patients in clinical remission [15, 16].

### Analysis

Demographics and clinical characteristics of the participants were summarized with descriptive statistics.

#### Structural validity

A confirmatory factor analysis (CFA) with weighted least square mean- and variance-adjusted estimator was performed to assess unidimensionality of the PROMIS-GI Scales. The distribution of answers for all items was reviewed. If a CFA could not be completed due to a highly skewed distribution of answers, response categories that were chosen by fewer than five patients were merged with an adjacent response category until a minimum of five answers were obtained in each response category.

To evaluate model fit comparative fit index (CFI), Tucker–Lewis Index (TLI), the root mean square error of approximation (RMSEA) and the standardized root mean square residual (SRMR) were used. Representative of a good fit was a CFI value > 0.95, RMSEA value < 0.08, TLI > 0.95, and a SRMR < 0.10 [17].

#### Construct validity: hypothesis testing

To assess the extent to which the DF-PROMIS-GI Scales are measuring the same or similar constructs as the scales of the three legacy instruments (IBDQ, IBS-QOL and GSRS), convergent validity was assessed. This was evaluated by calculating Pearson’s correlations of the DF-PROMIS GI Scale T-scores with the total scores of the disease specific instruments. According to COSMIN guidelines [18], hypotheses were formulated a priori regarding the expected correlations based on previous research (Table 1) [2]. A moderate to strong correlation was considered (r > 0.40) between the DF-PROMIS GI Scales and the three legacy instruments, based on the results of the original PROMIS GI development study. Convergent validity was considered to be adequate if at least 75% of the results were in accordance with the hypotheses.

**Table 1:** Hypotheses of PROMIS Gastrointestinal Symptom Scales with legacy measures

	GSRS reflux	GSRS indigestion	GSRS belly pain	GSRS diarrhea	GSRS constipation	GSRS total	IBD-Q	IBS-QOL
PROMIS gastroesophageal reflux	> 0.40^a^	> 0.40	> 0.40	< 0.40^b^	> 0.40	> 0.40	> − 0.40	< − 0.40
PROMIS disrupted swallowing	> 0.40	> 0.40	> 0.40	< 0.40	> 0.40	> 0.40	< − 0.40	< − 0.40
PROMIS diarrhea	< 0.40	> 0.40	< 0.40	> 0.40	< 0.40	> 0.40	> − 0.40	> − 0.40
PROMIS incontinence	< 0.40	< 0.40	< 0.40	> 0.40	< 0.40	> 0.40	> − 0.40	< − 0.40
PROMIS nausea and vomiting	> 0.40	> 0.40	> 0.40	< 0.40	> 0.40	> 0.40	> − 0.40	> − 0.40
PROMIS constipation	< 0.40	> 0.40	> 0.40	< 0.40	> 0.40	> 0.40	> − 0.40	< − 0.40
PROMIS abdominal pain	> 0.40	> 0.40	> 0.40	> 0.40	> 0.40	> 0.40	> − 0.40	> − 0.40
PROMIS gas and Bloating	> 0.40	> 0.40	> 0.40	> 0.40	> 0.40	> 0.40	> − 0.40	> − 0.40

*GSRS* gastrointestinal symptom rating scale, *IBDQ* Inflammatory bowel disease questionnaire, *IBS-QOL* irritable bowel syndrome-quality of life, *PROMIS* patient-reported outcomes measurement information system

^a^Pearson ‘s r of > 0.040 represent a moderate to strong correlation

^b^Pearson ‘s r of < 0.040 represent a weak correlation

IBM^®^ SPSS^®^ Statistics for Windows version 29.0., Armonk, NY was used for descriptive statistics and hypotheses testing. The R-package “lavaan (v0.6.14)” [19] was used for structural validity.

## Results

### Translation and cognitive debriefing

Eight PROMIS GI Scales were translated into Dutch–Flemish (DF), and all of them had translation issues to be resolved. Nineteen out of the 77 items (24.7%) were challenging for translation and required specific linguistic attention. The term ‘how much’ was used in 12 source items and was translated into ‘in welke mate’ (to what extent), to ensure consistency with previously translated PROMIS measures. Two source items use the phrase ‘make it to the bathroom’. After discussion this was translated as ‘bij het toilet kon zijn’ (get to the toilet). The term bathroom is not used in Dutch for going to the toilet, but for going to the shower instead. In the Scale Diarrhea the term loose is used three times, which in Dutch means ‘losse’. Since ‘losse’ is not a commonly used term to describe stool consistency, therefore ‘dunne’ (thin) was chosen. Two items of the Scale Gastrointestinal Disrupted Swallowing use the phrase ‘in your chest’. In Dutch, symptoms of dysphagia are explained as that food gets stuck or does not lower behind the breastbone. Therefore, the phrase ‘achter het borstbeen’ (behind the breastbone) was chosen.

Subsequently, the DF-PROMIS GI Scales were tested for comprehensibility in the Netherlands and Belgium. In total 20 respondents (10 from the Netherlands, and 10 from Belgium) participated in the interviews, of which 60% were men (n = 12) with an average age of 50.5 years (19–77). Five IBD patients were included, two IBS patients, three GERD/Barrett’s esophagus patients and ten people from the general population with no GI diseases. Ten minor changes in wording of the items were made after the interviews (Appendix 1). In addition, changes were made to the translations of the response options: ‘never’, ‘one day’, ‘2–6 days’, ‘once a day’, ‘more than once a day’. Particularly, the difference between ‘one day’ and ‘once a day’ was not clear in the first ten interviews. The translation was changed to: ‘nooit (never)’, ‘een keer tijdens de afgelopen 7 dagen (once in the last 7 days)’, ‘2–6 keer tijdens de afgelopen 7 dagen (2–6 times during the last 7 days)’, ‘vaak (eenmaal per dag) often (once per day)’, and ‘meer dan eenmaal per dag (more than ones per day)’.

The term breastbone is used in multiple Scales, but only in the Scale Gastrointestinal Reflux an image of the location of the breastbone is used for explanation. Respondents stated that adding the image also to the Scale Gastrointestinal Disrupted Swallowing would help them identify the location of the breastbone. This is particularly important for respondents who will not complete all GI Scales in the future. Therefore, the image was added to the DF-PROMIS GI Gastrointestinal Disrupted Swallowing Scales.

### Psychometric testing

The DF-PROMIS GI Scales and legacy instruments were completed by a total of 216 patients with GI conditions (IBD n = 95; IBS n = 50; GERD/Barrett’s esophagus n = 66, other GI disease = 2). The mean (SD) age was 54.8 (17.2) years, 50% were male, and 83.7% had a minimum of college education (Table 2).

**Table 2:** patients characteristics

Patients characteristics	n = 216 (%)
Male gender	109 (50.4)
Age in years, mean (SD)	54.8 (17.2)
Belgium/Flanders	74 (34.3)
Netherlands	142 (65.7)
*Diagnosis*	
Inflammatory bowel disease	96 (44.4)
Irritable Bowel Syndrome	51 (23.6)
Barrett’s esophagus/ GERD	67 (31.0)
Other GI condition*	2 (1.0)
*Education*	
High school graduate or less	24 (11.1)
Some college	88 (40.7)
Bachelor/ University graduate	93 (43.0)
Missing	12 (5.2)

Other GI conditions were: cirrhosis of the liver = 1, coeliac disease = 1

*GERD* gastro esophageal reflux disease, *SD* standard deviation

Scores of all the DF-PROMIS GI Scales and legacy instruments are shown in Table 3. The mean score of the DF-PROMIS GI Gas and Bloating Scale was above 50 (53.0), indicating that our patients reported more or more severe symptoms on average than the US general population. All other Scale mean scores were lower than 50, which means that the included patients scored fewer or less severe symptoms than the US general population.

**Table 3:** Scores DF-PROMIS GI Scales and legacy instruments

Questionnaires	Mean	(SD)
DF-PROMIS gastrointestinal reflux	45.7	(8.0)
DF-PROMIS gastrointestinal disrupted swallowing	46.3	(7.0)
DF-PROMIS gastrointestinal diarrhea	48.7	(8.8)
DF-PROMIS gastrointestinal bowel incontinence^a^	5.5	(2.6)
DF- PROMIS gastrointestinal nausea and vomiting	47.5	(8.0)
DF-PROMIS gastrointestinal constipation	49.8	(8.5)
DF- PROMIS gastrointestinal belly pain	49.7	(12.0)
DF-PROMIS gastrointestinal GI gas and bloating scale	53.0	(8.9)
IBD-Q	182	(29.2)
IBS-QOL	71.1	18.6
GSRS reflux	2.28	1.2
GSRS abdominal pain	1.67	1.0
GSRS indigestion	2.79	1.2
GSRS diarrhea	2.58	1.6
GSRS constipation	2.39	1.3
GSRS	2.48	1.0

*SD* standard deviation, *DF* Dutch Flemish, *PROMIS* Patient-reported outcomes measurement information system, *GSRS* gastrointestinal symptom rating scale, *IBDQ* inflammatory bowel disease questionnaire, *IBS-QOL* irritable bowel syndrome-quality of life

^a^The Health Measures version of the PROMIS Bowel incontinence Scale does not produce an IRT-based T-score. Therefore a summed scores was used (possible score range 4 to 20)

#### Structural validity

For the Scales DF-PROMIS GI Bowel Incontinence and Disrupted Swallowing, a CFA could be performed. For the other Scales the distribution of answers was highly skewed and a CFA could not be completed. After merging response categories in the Scales Reflux, Diarrhea, Constipation, Belly Pain and Gas and Bloating a CFA could be performed in these Scales. The data of the PROMIS Scale Nausea and Vomiting was still highly skewed after merging response categories and therefore CFA could not be performed.

The CFA for the Scales Gas and Bloating, Belly Pain and Bowel Incontinence showed a good fit (Table 4). The Reflux, Disrupted Swallowing, Diarrhea, and Constipation scales did not show a sufficient fit.

**Table 4:** Confirmative factor analysis

DF-PROMIS gastrointestinal scales	CFI	RMSEA	TLI	SRMR
Reflux*	0.463	0.135	0.356	0.170
Disrupted swallowing	0.871	0.068	0.806	0.057
Diarrhea*	0.905	0.135	0.842	0.064
bowel incontinence	0.999	0.068	0.999	0.013
Constipation*	0.664	0.131	0.553	0.116
Belly pain*	0.998	0.030	0.967	0.020
Gas and bloating*	0.952	0.071	0.942	0.067

*DF* Dutch Flemish, *PROMIS* Patient-Reported outcomes measurement information system, *CFI* Comparative fit index, *RMSEA* root mean square error of approximation, *TLI* Tucker–Lewis index, *SRMR* standardized root mean square residual

*Distribution of response categories was highly skewed, and responses were merged. The complete overview of the merged categories is descripted in Appendix 2

#### Construct validity: hypothesis testing

Table 5 summarizes the correlations between the DF-PROMIS GI T-scores and the legacy instrument scores. Six out of eight Scales (Reflux, Diarrhea, Bowel Incontinence, Nausea and Vomiting, Belly Pain, and Gas and Bloating) showed sufficient convergent validity with more than 75% of hypothesis confirmed.

**Table 5:** Correlations of DF-PROMIS gastrointestinal Scales with legacy measures

DF-PROMIS gastrointestinal scales	GSRS reflux	GSRS Indigestion	GSRS belly pain	GSRS diarrhea	GSRS constipation	GSRS total	IBD-Q	IBS-QOL	Confirmed (%)
Reflux	**0.55**	**0.47**	**0.47**	**0.19**	0.33	**0.53**	− 0.33	− **0.38**	75
Disrupted Swallowing	**0.42**	0.30	0.36	**0.15**	0.29	0.38	**− .019**	**− 0.26**	50
Diarrhea	**0.06**	0.37	**0.39**	**0.79**	**0.21**	**0.56**	− **0.66**	− **0.40**	88
Bowel incontinence	**0.06**	**0.20**	**0.18**	0.45	**0.19**	0.30	− **0.46**	− **25**	75
Nausea and vomiting	0.31	**0.48**	**0.64**	**0.34**	0.33	**0.58**	− **0.54**	− **0.41**	75
Constipation	**0.20**	**0.42**	0.34	**0.17**	**0.78**	**0.49**	− 0.27	− 0.29	63
Belly pain	0.31	**0.63**	**0.65**	**0.57**	**0.52**	**0.75**	− **0.75**	− **0.53**	88
Gas and Bloating	0.33	**0.74**	**0.51**	0.35	**0.49**	**0.65**	− **0.48**	− **0.47**	75

Pearson correlation coefficients were calculated. Results in accordance with a priori hypothesized correlations are bold

*GSRS* gastrointestinal symptom rating scale, *IBDQ* Inflammatory bowel disease questionnaire, *IBS-QOL* Irritable bowel syndrome-quality of life, *PROMIS* patient-reported outcomes measurement information system

Although only five out of eight hypothesis of the PROMIS Scale Constipation were consistent with the hypotheses, a high correlation (0.78) was found with de GSRS constipation scale. Low correlations were found for the PROMIS Disrupted Swallowing Scale and only four out of the eight hypothesis good be confirmed.

As Table 5 shows, high correlations were found (*r* 0.56–0.79) between scales measuring the same construct.

## Discussion

With this study, the PROMIS GI Symptom Scales were translated in Dutch–Flemish and their psychometric properties, structural validity and construct validity, were evaluated. The translation was performed using a rigorous, standardized methodology. The FACIT translation methodology was developed based on comprehensive research in the HRQOL field to ensure that the translations are conceptually equivalent to the English source and are rendered in a language that is culturally acceptable and relevant to the target audience. Nineteen out of the 77 items (24.7%) were challenging for translation and required specific linguistic attention. Those items were discussed between the translators, after which consensus was achieved. Subsequently, in the cognitive debriefing phase, ten minor changes in the wording of the items were made. There were no cross-cultural issues identified. In general, patients stated that they had no difficulty understanding the DF-PROMIS GI items, and could use these items to self-report their GI symptoms. We finally succeeded in developing one universal Dutch–Flemish translation for all 77 items.

To our knowledge, this is the first study investigating the psychometric properties of the PROMIS-GI Scales v1.0 outside the US. CFA analysis could initially only be performed on the two Scales Incontinence and Disrupted Swallowing. The highly skewed data on all of the other Scales were probably due to the expected missings, the low variation in reported symptoms, and patients being more likely to have fewer or less severe symptoms. After merging response categories, a CFA analysis for the majority of the Scales could be performed. The Scale Nausea and Vomiting was still highly skewed after merging the responses and therefore CFA could not be performed on this Scale. Remarkably, Spiegel et al. [3] were able to run CFA without merging response categories. This may be explained by the fact that our respondents reported fewer and less severe symptoms, resulting in skewed data with more scores of one or two. Also the variation in responses was higher in the sample of Spiegel et al., compared to our sample.

A good fit was found for three Scales: Gas and Bloating, Bowel Incontinence, and Belly Pain. This means that these Scales are considered unidimensional and that there is a single latent trait underlying the responses. Poor fit was found for the Scales Reflux, Disrupted Swallowing, Diarrhea, and Constipation, in contrast to the findings of the original development study. A possible explanation for this might be the skewed data or the heterogeneous sample. Alternatively, (some of) the concepts aimed to be measured by these scales might be more multidimensional in the Dutch and Belgian cultures. This should be tested in a future study.

Construct validity was considered sufficient for six out of eight DF-PROMIS GI Scales. For the Bowel Incontinence and Disrupted Swallowing Scales less than 75% of the hypothesis could be confirmed. The hypotheses were predefined based on the first and only study validating the PROMIS GI Scales. In line with the original PROMIS-GI data, this study showed high correlations between the DF-PROMIS GI Scales and subscales of the legacy instruments measuring the same constructs. For example, the DF-PROMIS GI Diarrhea Scale showed a Pearson correlation of 0.79 with the GSRS diarrhea subscale, which support the validity of the GI Scales.

Interestingly, mainly weak correlations were found between the Disrupted Swallowing Scale and the legacy instruments. This may be explained by the fact that the legacy questionnaires do not contain questions about difficulties with swallowing or passage of food through the esophagus, although higher correlations were found in the original development study. This may be explained by the fact that the participants in Spiegel's study reported more symptoms, thus making overlap of different GI symptoms more likely. It is well known that some patients with FGID can have more than one FGID. This overlap could affect the primary symptomatology of different disorders [20–22].

Only 63% of the hypothesis for the Constipation Scale could be confirmed. The a priori defined hypotheses were entirely based on the work of Spiegel et al. [3]. Surprisingly, Spiegel et al. found moderate correlations between Scales that were not measuring the same construct (e.g. PROMIS GI Constipation versus IBD-Q r = 0.54). In general, and in contrast to the present study, Spiegel et al. reported more moderate correlations (0.40–0.70) between the PROMIS GI Scales and the legacy instruments IBDQ and IBS-QOL. Possibly this was caused by the fact that the patients included in the study of Spiegel et al. reported more and more severe symptoms than the patients in the current study. This may have caused that there was more overlap in the GI symptoms present, and therefore higher correlations were found for the study of Spiegel et al. compared to the current study. There was also more variation in T-scores in the sample of Spiegel et al., which leads to higher correlations.

The majority of the PROMIS Scales use a T-score metric with a mean score of 50 (representing the mean score of the US reference population) and a standard deviation of 10. A remarkable finding of this study was that the T-scores of all Scales except the DF-PROMIS Gas and Bloating Scale were below 50. This seems to show that the enrolled patient group as a whole (IBD, IBS and reflux) reported fewer and less severe symptoms than a US general population. Another explanation could be the presence of differential item functioning (DIF).

Additional research can determine whether there is DIF between US and DF patients within the PROMIS GI Scales, after allowing for overall subgroup differences in that scale.

When we analyzed the disease groups separately, we found that only the IBS patients reported an average T-score above 50 on four out of the eight Scales (Diarrhea, Constipation, Belly Pain and Gas and Bloating). IBD patients in clinical remission generally report a score of 170 or higher on the IBD-Q [15]. The included Dutch and Belgian patients in the present study scored an average of 182, which suggest that we mainly included patients in remission. However, one would expect IBD patients in remission to report more GI symptoms than a generic population. Previous research found that IBD patients in remission often experience symptoms similar to those of IBS [23].

When comparing the mean T-scores of the DF-PROMIS GI with the study from Spiegel et al. who included US patients with similar GI diseases, it is also notable that the American population with GI diseases reported relatively low T-scores (e.g. 51–57). However, in contrast to T-scores found in the present study, always slightly above 50. A possible explanation for the discrepancies may be the differences in experiencing GI symptoms between countries. A world-wide study on the prevalence of FGID showed that persons living in the US reported a higher percentage of any FGID in comparison to persons living in the Netherlands (39.9 in the US versus 30.6 in the Netherlands). Specifically, the US population reported double the amount of functional dyspepsia as compared to Dutch and Belgium residents. This raises the question if the interpretation of a T-score of 50 as the mean score of the general population would also be applicable to the Dutch population. To determine the true differences between the Dutch and US (norm) population, further research should be undertaken to investigate T-scores in a Dutch general population. Another possible explanation for the differences in observed T-scores between the two studies is the difference in disease severity. There were no mean scores described of the legacy instruments IBS-QOL, IBDQ and GSRS in the article of Spiegel et al. As a result, it is unclear whether the study populations are comparable.

A limitation of our study is that our sample may not accurately reflect the population of Dutch and Belgian patients with a GI condition, considering the low T-scores. Another limitation is that we only assessed convergent validity and did not have data to test discriminant validity. Another limitation is the highly skewed data of all of the PROMIS GI Scales, indicating that the patient sample was not very heterogeneous. These have negatively influenced the outcomes of the CFA analysis and may also have influenced the correlations with the legacy instruments. Since the present study did not assess other psychometric properties such as discriminant validity, test–retest reliability and cross-cultural validity, for the population of Dutch and Belgian patients with a GI condition, nor the Dutch and Belgian general population, future research should address these properties. Furthermore, it is important to obtain both T-scores of the Dutch and Belgian general population.

In conclusion, The PROMIS GI Symptom Scales were successfully translated into Dutch–Flemish. The findings suggest a sufficient structural validity for the PROMIS GI Scales Bowel Incontinence, Gas and Bloating and Belly Pain. Construct validity was considered sufficient for the Scales Gas and Bloating, Incontinence, Nausea and Vomiting, Reflux, Belly Pain, and Diarrhea. The DF-PROMIS GI Symptom Scales are available on request from the Dutch–Flemish PROMIS National Center (www.dutchflemishpromis.nl).

## Data Availability

The datasets used and/or analyzed during the current study is available from the corresponding author on reasonable request.

## Appendix 1: Adjustments made after the debriefing interviews of the translated PROMIS gastro intestinal scales

**Table:**

Item Number	Source item	Dutch version English equivalent	Final Dutch version	Reason for adaptation
GISX45	How often did you have bowel incontinence—that is, have an accident because you could not make it to the bathroom in time?	How often did you have bowel movements—that is to say soil your underwear because you did not get to the toilet in time?	Hoe vaak had u ongewenst ontlasting verlies—dat wil zeggen bevuilde u het ondergoed omdat u niet op tijd bij het toilet kon zijn?	The term “darmincontinentie” is not clear. After adjusting to “ongewenst ontlasting verlies” no more comments were made. The term "een ongelukje krijgen” was not clear and could also be interpreted as having an accident (falling). After adjustment to “soiling your underwear,” no more comments were made
GISX66	How much did you usually strain while trying to have a bowel movement?	To what extent did you have to press hard when you tried to get bowel movement?	In welke mate moest u hard persen wanneer u probeerde ontlasting te krijgen?	Three out of 10 respondents recommended to delete the word ‘gewoonlijk’. After adjustments no comments in the next ten interviews were made
GISX68	How often did you feel pain in your rectum or anus while trying to have bowel movements?	How often did you feel pain in your last part of the bowel or anus while trying to have bowel movements?	Hoe vaak voelde u pijn aan uw laatste stukje van de darm of anus wanneer u probeerde ontlasting te krijgen?	Nine out of 10 respondents did not know what “rectum” means. And indicated that this is probably the same as the anus. After adjusting to “laatste stukje van de darm,” no comments were made in the last ten interviews
GISX42	How often did you feel like you needed to empty your bowels right away or else you would have an accident?	How often did you feel like you needed to empty your bowels right away or else you would soil your underwear	Hoe vaak had u het gevoel dat u uw darmen meteen moest legen, anders zou u uw ondergoed bevuilen?	The term ‘een ongelukje krijgen’ was not clear and can also be interpreted as getting into an accident. After adjustment to ‘uw ondergoed bevuilen’ no comments were made
